# Wood-Based Materials in Building

**DOI:** 10.3390/ma16082987

**Published:** 2023-04-09

**Authors:** Dorota Dukarska, Radosław Mirski

**Affiliations:** Department of Mechanical Wood Technology, Faculty of Forestry and Wood Technology, Poznań University of Life Sciences, 60-627 Poznań, Poland

Wood is a widely used building material. It is characterized by a high strength-to-weight ratio, predictable fire behavior, good performance in seismic zones, and it is easy to use in the construction of prefabricated buildings. In addition, wood reduces energy consumption during construction and reduces a building’s overall environmental impact, has lower embedded energy compared to steel and concrete, and has a positive effect on a building’s carbon footprint [1]. The use of wood in building constructions is limited by its tendency to crack, twist fibers, and it can be difficult to obtain the right dimensions and shapes for use. As a result, it is being replaced by new-generation wood-based materials. Therefore, the aim of this Special Issue is to present the latest knowledge and current trends in the production of innovative wood-based materials, including composite materials for modern wood constructions, and to provide new solutions to ensure better strength parameters and safer wood constructions. Such solutions include the reinforcement of ashlar glulam beams, proposed by Wdowiak-Postulak [2], using carbon fiber reinforced plastic (CFRP) cords and carbon laminates made of carbon fibers embedded in an epoxy resin matrix. Two types of reinforcement were tested, i.e., internal strengthening, in which carbon cords are placed into cut grooves in the last and penultimate lamella, and an external surface of near-surface mounted carbon laminates, which are glued to the bottom surface of the beam to reinforce the laminated ashlar beams. The effectiveness of the reinforcement of the beams was evaluated in a four-point bending test. It was found that reinforcing the glulam beams of ashlar with carbon cords increases their load-bearing capacity by about 36%, and with carbon cords and carbon laminates by about 45%. At the same time, this type of beam reinforcement reduces the amount of displacement of the timber materials. From a practical point of view, it is important that this type of beam reinforcement is used in the construction of new structures, as well as the renovation of existing ones.

For multi-story floors, glulam-concrete composite beams (GCC) have been used for years, in which bending tensile forces are mainly carried by laminated wood, and compressive forces by concrete. Compared to typical glulam beams, GCC beams are characterized by, among other things, better load-bearing capacity, flexural strength and stiffness, better sound insulation, and greater thermal mass [3]. Du et al. [4], conducting experimental studies and finite element modeling, determined the properties of GCC beams depending on the height of the glulam beam, the spacing of the shear connectors, the thickness of the timber board interlayer, and the thickness of the concrete slab. It was shown that the failure mechanism of this type of beam is the combination of bend and tensile failure in the glulam beam, and that the weakening of interfacial interaction leading to a sharp increase in slip in the interfacial region occurs at a load corresponding to 28% of the maximum load. Increasing the height of the glulam beam results in a significant increase in the flexural capacity and stiffness of the GCC beams. On the other hand, increasing the spacing of the shear connectors decreases the ultimate bearing capacity and bending stiffness of the beams. The timber boards used as formwork for pouring concrete, placed on top of the glulam beam, have no significant effect on the flexural performance of GCC beams. It is clear that the bending bearing capacity and flexural stiffness of the composite beams increase as the thickness of the concrete slab increases. It is noteworthy that the finite element method and numerical simulation used by the authors make it possible to accurately predict the failure mode and change characteristics of GCC composite beams during the loading process.

The permanent preservation of the form and safety of wooden constructions is ensured not only by the structural elements with adequate load-bearing capacity and strength but also by the properties of connectors and connections of individual components. Among the most common connectors in wooden constructions are dowel-type fasteners, which include screws, dowels, and bolts. According to Johanides et al. [5,6], in order for them to perform their function, it is necessary to know their mechanical behavior under load and the relationship between load and slip, stress distribution, and possible different failure modes. Therefore, the authors conducted a series of tests on the basis of which they determined the load-carrying capacity, and the rotational stiffness of a semi-rigid connection of a rung and two stands using dowel-type mechanical fasteners. These tests were further validated through numerical models. Two types of fasteners were evaluated, i.e., those made from a combination of bolts and dowels, and those made from high-strength fully threaded screws. In the first case, the load-carrying capacity and rotational stiffness were found to be higher than the values estimated according to the standard for the ultimate load condition during the entire loading process. The second type of connection from fully threaded screws also showed a higher load carrying capacity compared to the design capacity, but its rotational stiffness did not reach values higher than those estimated for the ultimate limit state for the load level corresponding to 80% of the ultimate limit state. Therefore, as the authors concluded, both types of connections are safe and reliable until the ultimate limit state is reached.

An important aspect of current research is numerical modeling, which is an excellent tool for understanding the behavior of joints in wood constructions [5,6]. The numerical analysis of single-step joints, applied by Braun et al. [7], made it possible to satisfactorily determine the stiffness and predict the forces at the onset of local failure of single- and double-step joints. In doing so, it was found that the prediction was more accurate for single-step joints, due to the fact that the model was calibrated for this type of joint, and that the joint itself is less susceptible to geometric inaccuracies. The developed model is recommended for the future, nondestructive testing of various types of wood joints to estimate their stiffness and failure mode.

An innovative solution for connecting light timber-framed roof elements has been proposed by Islam et al. [8]. The apex connection developed by the authors is expected to reduce the workload both at the construction site and at the roof panel manufacturing plant, in order to allow multiple panels to be lifted at the same time, and to streamline their transportation and installation at the site. In addition, the fastener itself is collapsible and has a self-locking mechanism that eliminates the installation steps of the panel connection on-site. To demonstrate the proposed solution, the authors prepared a 3D printout of the developed joint, which shows its effectiveness in providing a folding and unfolding mechanism as well as a self-locking mechanism for a lightweight panel roof. In addition, a finite element analysis was carried out to determine the strength requirements of this connection according to different load cases. The results of this analysis as well as the prepared 3D model indicate that it is useful and significantly improves the transportation and installation of the panel roof, mainly by providing a mechanism for its folding before installation. They also demonstrated that it can withstand the load in its unfolded state at service.

The safety of wood construction relies on proper construction technique, the quality of the engineered wood, and fire resistance. The fire safety of the structure is one of the basic requirements to be met when designing, constructing, and using a wooden house. For this reason, flame retardants are used for wood and wood-based materials, which are designed to, among other things, reduce the spread of fire, weight loss, and the rate of heat release. However, the use of flame retardants affects the mechanical properties of the wood. Grześkowiak et al. [9] determined the effect of wood flame retardants on the compressive strength and elastic modulus of wood as a function of accelerated aging time. The wood was treated with solutions of chemical compounds included in the flame retardant formulations, i.e., monoammonium phosphate, boric acid, sodium tetraborate (borax), urea, monoammonium sulphate and diammonium phosphate, as well as commercially available formulations. In addition, to determine the progressive changes in the wood over time, a proprietary accelerated aging cycle was developed to simulate conditions in temperate climates. The process included 0, 8, and 16 cycles, and each cycle consisted of the following phases: heating at 130 °C for 24 h, then freezing at −15 °C for 24 h, reheating to 130 °C for 24 h, conditioning at 40–45 °C and 90% relative humidity for 24 h, and refreezing at −15 °C for 24 h. Of the non-aged wood samples tested, wood treated with urea, boric acid borax, and monoammonium phosphate showed the lowest compressive strength. A significant decrease in the modulus of elasticity was also observed for the last compound. In contrast, after a full aging process of 16 cycles, wood treated with urea, diammonium phosphate, and boric acid showed the highest compressive strength values. In the case of elastic modulus, the best results were obtained using monoammonium phosphate. Protecting the wood with a commercial formulation gave positive results, but only for a maximum of eight aging cycles.

One of the research directions presented in this Special Issue is the possibility of using wood by-products from primary wood processing in the production of wood-based and insulation materials for the wider construction industry. In the sawmill industry, during the primary processing of wood raw material, in addition to losses due to desorption changes, material losses of up to 50% of the wood raw material originally intended for production are also generated [10]. These losses primarily involve wood chips, sawdust, and bark, which must be managed. Mirski et al. [11] demonstrated the possibility of managing ground bark and sawdust from pine roundwood processing lines as a partial substitute for wood chips during the production of wood-based boards glued with urea–formaldehyde (UF) resin. In the produced boards, the proportions of wood chips to bark were 70:30, 60:40, and 50:50, while that of sawdust was 70:30. Regarding the results of the study, the authors showed that the most homogeneous structure and the most favorable properties are characterized by boards in which the weight ratio of wood chips to finer particles (i.e., sawdust or bark) is 70:30. They also found that the partial substitution of sawdust for bark increases the homogeneity of the board cross-section, and contributes to a significant reduction in formaldehyde emissions and water absorption. However, a potential limitation of its application in industrial practice is the variability of the chemical composition of the bark. Therefore, further research is needed, taking into account the influence of factors such as wood species, habitat, age, size, and quality of the debarked log on board properties.

The bark has a unique chemical composition and contains numerous organic compounds such as tannins, lignin, cellulose, catechins, gallocatechins, flavonoids, and proanthocyanidins [12,13,14]. For this reason, Walkiewicz et al. [15] used the powdered bark of various tree species as UF resin fillers in the process of plywood production. Birch, beech, maple, pine, and spruce bark were considered. Replacing the traditional filler (i.e., rye flour commonly used in the plywood industry) with maple bark did not affect the bond quality of the plywood produced with it. In other cases, a significant decrease in tensile strength values was noted, especially in the case of spruce bark. Despite these decreases, the obtained values still exceeded the normative requirements, i.e., they were above 1 N/mm^2^. The decrease in the bond quality of plywood glued with resin with the addition of different bark species is due to its chemical composition, which can affect the resin curing process and lower the pH of the adhesive mixture. The consequence of this can be the occurrence of resin pre-curing and a decrease in the strength of the adhesive bond. In addition, spruce bark causes a decrease in the elastic modulus of plywood and its bending strength, especially in the perpendicular direction. However, the presence of lignin as well as tannins in the bark contributed to a favorable reduction in formaldehyde emissions, which was clearly observed for birch, beech, maple, and pine bark. Only in the case of spruce bark was a deterioration in the hygienic quality of the plywood produced.

By-products from the wood industry have also become a source of fillers that can be used in the production of polymer-based composites. Dukarska et al. [16] showed that pine sawdust is a material with a high application potential in the production of polyurethane (PUR) composite foams. The authors determined the effect of modification of a rigid PUR foam with a closed-cell structure with various amounts of sawdust, with particle sizes in the range of 0.315–1.25 mm, on the kinetics of the foam foaming process, its structure, and selected functional properties. It was shown that the introduction of up to 10% by weight of sawdust into the structure of foams does not significantly affect the course of the foaming process, while such an amount of filler allows them to reduce their thermal conductivity coefficient and significantly reduce their brittleness, while maintaining the required dimensional stability. This is accompanied by a slight decrease in the compressive strength of the foams, a decrease in their flexural strength, and an increase in water absorption. However, despite the slight decrease in the values of the above parameters, composite foams containing up to 10% sawdust are characterized by favorable properties comparable to rigid PUR foams currently available on the market. The introduction of larger amounts of sawdust into the polyurethane matrix, i.e., at the levels of 15% and 20%, results in serious changes in the cellular structure of PUR foams, which, as a consequence, leads to a significant decrease in almost all the studied physical and mechanical parameters. Thus, as the authors propose, rigid PUR foams made with a 10% addition of pine sawdust can be used as thermal insulation materials in the construction industry, for example for door insulation. However, they can also be used in the refrigeration and heating industries, for example, for the insulation of refrigerated furniture or boilers. The significantly reduced brittleness of this type of foam also allows it to be used in the production of usable items such as ceiling and wall decorations.

The current technological and social trends require the construction sector to implement the principles of sustainable development. An important element of this concept is the principle of wooden construction based mainly on wood and wood-based materials, which counteracts climate change by storing carbon dioxide in wood and reducing the consumption of high-emission materials such as concrete and steel. The introduction of new and innovative solutions improves the energy efficiency of buildings, their functionality, and reduces their negative impact on the natural environment. The works presented in this Special Issue exemplify the current directions of research in modern wooden construction in accordance with the concept of sustainable development.
